# The Influence of Trait and State Creative Self-Efficacy on Creative Behavior: An Experimental Study Using False Feedback

**DOI:** 10.3390/bs15010018

**Published:** 2024-12-28

**Authors:** Rui Tao, Haoman Zhang, Li Geng, Yu Li, Jiang Qiu

**Affiliations:** 1 Key Laboratory of Cognition and Personality (SWU), Ministry of Education, Chongqing 400715, China; hututu@email.swu.edu.cn (R.T.); crystalbingtang@126.com (H.Z.); swugengl@email.swu.edu.cn (L.G.); 2 Faculty of Psychology, Southwest University (SWU), Beibei District, Chongqing 400715, China

**Keywords:** creative self-efficacy, trait self-efficacy, state self-efficacy, false feedback, creative behavior

## Abstract

This study explores the relationship between creative self-efficacy and creative behavior by modulating state-level creative self-efficacy through false feedback to enhance creative performance. In Study 1, 1539 college students completed the Alternative Uses Task (AUT) to measure performance-based creativity and the Creative Achievement Questionnaire (CAQ) to assess self-reported creative achievements. The Big Five personality traits and curiosity served as covariates. Regression and correlation analyses were conducted to examine the relationship between trait-level creative self-efficacy and both creative performance measures. Experiment 2 employed a 2 × 2 between-subjects design to test the effects of false feedback (positive vs. negative) and trait-level creative self-efficacy (high vs. low) on state-level creative self-efficacy and creative behavior, using the same covariates. In Study 1, creative self-efficacy was positively correlated with both AUT and CAQ, with stronger associations for CAQ. Experiment 2 found that false feedback significantly affected state-level creative self-efficacy and the originality of creative behavior. Changes in state-level creative self-efficacy were positively correlated with originality. This study emphasizes the role of both trait and state-level creative self-efficacy in influencing creative behavior. It offers insights for enhancing creativity through feedback, with implications for educational and workplace settings.

## 1. Introduction

Creativity is widely recognized as a multifaceted construct that encompasses cognitive, emotional, environmental, and cultural dimensions. At its core, creativity is commonly defined as the ability to produce novel and appropriate works, reflecting its dynamic interplay among individual traits, cognitive processes, and external influences (Glăveanu & Kaufman, 2020; Rhodes, 1961; Runco, 2007; Sternberg & Karami, 2022). In this study, we explore creativity by focusing on its behavioral manifestations, examining how both internal traits and situational factors shape creative behavior (Beaty et al., 2016; Beaty et al., 2019). Given the significant impact of creativity on both individual and societal progress, researchers have been dedicated to identifying the factors that influence individual creative behavior. Enhancing creativity is critical not only for personal development but also for driving innovation across industries, fostering problem-solving capabilities, and addressing complex global challenges.

Creative self-efficacy, a key concept in creativity research, refers to an individual’s belief in their capacity to generate creative ideas or solutions (Puente-Díaz, 2016; Tierney & Farmer, 2002). This construct is divided into two types: trait creative self-efficacy, which reflects a stable and enduring belief in one’s creative abilities, and state creative self-efficacy, which is more dynamic and influenced by situational factors such as the environment and emotional states (van Diemen et al., 2020). Trait creative self-efficacy underpins long-term creative pursuits, such as an artist’s sustained confidence in their craft, or a scientist’s enduring belief in their innovative potential. Conversely, state creative self-efficacy can be observed in temporary contexts, like a student feeling empowered to propose novel ideas during a classroom activity after receiving encouraging feedback. These two types of self-efficacy are particularly relevant in the Four C Model of Creativity: state creative self-efficacy can drive mini-c creativity, representing personal insights and novice-level creative expressions, while trait creative self-efficacy supports Pro-C and Big-C creativity, which correspond to professional and eminent levels of achievement (J. C. Kaufman & Beghetto, 2009; Helfand et al., 2016).

Despite this theoretical and empirical support, research findings regarding the relationship between trait creative self-efficacy and creative performance are inconsistent (Haase et al., 2018; Intasao & Hao, 2018; Puente-Díaz, 2016; Richter et al., 2012; Simmons et al., 2014). Specifically, these mixed findings often reflect differences in the magnitude of correlations observed between creative self-efficacy measures and two distinct types of creativity assessments: self-reported achievement measures (e.g., the Creative Achievement Questionnaire) and performance-based creativity measures (e.g., the Alternate Uses Task). When self-reported achievement measures (such as the Creative Achievement Questionnaire) are used to measure individuals’ creative behavior, the relationship between trait creative self-efficacy and creative performance is typically significant. In contrast, when performance-based creativity measures (such as the Alternate Uses Task) are used to measure creative behavior, the relationship between the two is often not significant or only marginally significant. This discrepancy may stem from the tendency of individuals with higher creative self-efficacy; there may be a tendency to embellish their creative performance (e.g., due to memory preferences), leading to a higher correlation between the two in self-reported achievement measures. However, performance-based creativity measures are more likely to capture individuals’ actual creative behavior levels, which may result in a lower correlation between creative self-efficacy and creative performance. Additionally, the observed high correlation between creative self-efficacy and the CAQ may also stem from common method bias, where shared method variance inflates correlations (Podsakoff et al., 2003). Incorporating performance-based creativity measures alongside self-report scales can enhance data diversity by observing behavior in addition to self-reported information, partially addressing common method bias.

Recent research on trait creative self-efficacy has advanced considerably, while research on state creative self-efficacy remains less explored. According to Bandura’s self-efficacy theory, trait creative self-efficacy is shaped by cumulative experiences and reflects an individual’s stable belief in their creative abilities. In contrast, state creative self-efficacy is more reactive and influenced by immediate contextual factors, such as external evaluations and feedback (Eden & Aviram, 1993). This distinction has been supported by previous empirical studies, which demonstrate that while trait creative self-efficacy provides a stable foundation for creative behavior, state creative self-efficacy can dynamically influence performance in specific situations (Camargo et al., 2020). Research has shown that manipulating state cultural self-efficacy, such as through positive feedback, can significantly improve short-term creative output (Camargo et al., 2020). This suggests that state creative self-efficacy can serve as a tool for overcoming short-term creative challenges, while trait self-efficacy remains more resilient to immediate contextual changes. Importantly, trait creative self-efficacy is not immune to external influences but develops over time through accumulated experiences, while state self-efficacy is more sensitive to the immediate context.

In addition to differences in measurement methods, the discrepancies in research findings on the relationship between trait creative self-efficacy and creative behavior may also be influenced by other interfering factors. Trait creative self-efficacy is influenced by various factors, including openness, extraversion, and conscientiousness from the Big Five personality traits, all of which are considered potential covariates of individuals’ creative self-efficacy (Karwowski, 2014; Karwowski et al., 2013; Puente-Díaz & Cavazos-Arroyo, 2017). Although the Big Five personality traits, curiosity, and creative self-efficacy are all related to creativity (Furnham & Bachtiar, 2008; Hass et al., 2019; S. B. Kaufman, 2013; Schutte & Malouff, 2020a, 2020b), current research, often limited by small sample sizes, rarely controls for these potential covariates when exploring the relationship between creative self-efficacy and creative behavior. Therefore, future research should further explore the relationship between trait creative self-efficacy and creative behavior by incorporating larger sample sizes and multiple covariates. This research direction is crucial for improving the validity of relevant studies and addressing the limitations in the current understanding of the relationship between creative self-efficacy and creative behavior.

The primary goal of this study is to explore the feasibility of influencing creative behavior by modulating creative self-efficacy. While creative self-efficacy has long been regarded as an individual trait closely associated with creativity, studies on the relationship between creative self-efficacy and creative behavior have yielded mixed results (Haase et al., 2018; Intasao & Hao, 2018; Puente-Díaz, 2016; Richter et al., 2012; Simmons et al., 2014). Specifically, these mixed findings often reflect differences in the magnitude of correlations observed between creative self-efficacy measures and two distinct types of creativity assessments: self-reported achievement measures (e.g., the Creative Achievement Questionnaire) and performance-based creativity measures (e.g., the Alternate Uses Task). Therefore, foundational research is needed to reassess this relationship, providing a theoretical basis for applied research aimed at enhancing creative behavior through the modulation of creative self-efficacy. The specific objectives of the study are as follows: (1) to assess the relationship between trait creative self-efficacy and creative behavior while accounting for covariates such as personality traits and curiosity, and (2) to examine the potential for enhancing individual creative performance by modulating state creative self-efficacy.

## 2. Methods

### 2.1. Participants

This study comprised two participant samples. Sample 1 consisted of participants from the Behavioral Brain Research Project of Chinese Personality (BBP), recruited through both online advertisements and offline questionnaire distribution. The recruiting program and exclusion procedures for these large investigations were described in detail elsewhere (L. He et al., 2021). A total of 1539 participants with complete data were included, ranging in age from 18 to 27 years (M = 19.71, SD = 1.53), with 1036 females and 503 males. All participants had no history of psychiatric or neurological disorders, nor any history of substance abuse. Each participant signed a written informed consent form and was compensated for their participation.

Participants in Sample 2 were similarly recruited through online advertisements and offline questionnaire distribution. A total of 90 right-handed, mentally healthy participants were initially included (M = 19.56, SD = 1.26). However, 5 participants were excluded due to incomplete or nonsensical responses to key questions, such as inserting random numbers or providing irrelevant answers. One additional participant completed only the first questionnaire and did not finish the second. After excluding these 6 participants, 84 participants remained, comprising 13 males and 71 females.

### 2.2. Materials

#### 2.2.1. Assessment of Creative Self-Efficacy

We utilized the Creative Self-Efficacy (CSE) subscale of the Short Scale of Creative Self (SSCS) to measure creative self-efficacy (Karwowski et al., 2018), which consists of six items (e.g., “I believe I can handle tasks requiring creative thinking”). Each item is rated on a 5-point Likert scale (1 = “definitely not true”, 5 = “definitely true”). The Chinese version of the CSE subscale, adapted and validated by W.-J. He and Wong (2021) in China’s Hong Kong, demonstrated robust psychometric properties, including excellent internal consistency reliability and evidence for construct validity through confirmatory factor analysis. The internal consistency reliability for the CSE subscale in this study was 0.892.

State creative self-efficacy was measured using a single-item self-report measure. Participants rated their confidence in their ability to generate novel answers (“I believe I can provide novel answers”) on a 5-point Likert scale. The use of a single item captures participants’ momentary confidence effectively, as supported by prior evidence demonstrating the validity and predictive utility of single-item measures in assessing psychological constructs (Hoeppner et al., 2011).

#### 2.2.2. Assessment of Creative Behavior

In Study 1, creative behavior was assessed using the Alternative Uses Task (AUT) and the Creative Achievement Questionnaire (CAQ). The overall creativity score for the AUT, ranging from 0 to 10, was directly rated by evaluators. The evaluators provided a holistic assessment based on the fluency of responses (number of valid responses), originality (novelty of responses), and flexibility (diversity of response categories). The inter-rater reliability for the overall creativity scoring was good (ICC = 0.819, Two-Way Mixed Effects Model, Consistency). In Study 1, the objects used for the AUT were “Newspaper” and “Straw”. The CAQ was used to assess creativity by evaluating creative achievements across various domains (e.g., visual arts, music, and science). The CAQ has shown high reliability and validity in assessing creative achievements among Chinese adults (Wang & Zhang, 2020), making it suitable for this study.

In Experiment 2, in addition to the AUT, a Creative Metaphor Task (CM) was employed to reduce the potential influence of participants’ familiarity with standard creativity tests. The CM task required participants to generate novel metaphorical meanings for the presented words. The scoring dimensions included the following: (a) fluency, referring to the number of valid responses; (b) originality, defined as the creativity and rarity of the responses, with each response rated on a scale of 1 to 5, and considering both the quantity and quality of the responses, the overall originality score for each item was directly evaluated by the raters on a scale of 0 to 10; and (c) flexibility, referring to the number of different categories of responses. In this experiment, “brick” and “cup” were used as objects in the AUT, while “eggshell” and “waterfall” were used as words in the CM task. Four raters evaluated the responses, and the final score for each participant was the average of the four ratings. The study primarily focused on three dimensions—originality (inter-rater reliability coefficient = 0.725), fluency (inter-rater reliability coefficient = 0.925), and flexibility (inter-rater reliability coefficient = 0.767)—to explore how different types of creative self-efficacy and feedback influence the performance across these dimensions of creative behavior. Inter-rater reliability was calculated using a Two-Way Mixed Effects Model with a Consistency definition (Ten Hove et al., 2022; Koo & Li, 2016).

#### 2.2.3. Assessment of Personality

Personality assessment was conducted using the Big Five Personality Inventory (Costa & MacCrae, 1992). This questionnaire, based on the Big Five personality theory, consists of 240 items that assess five dimensions: openness, neuroticism, agreeableness, conscientiousness, and extraversion. Each dimension is measured by 48 items, with responses rated on a 5-point Likert scale ranging from “strongly disagree” to “strongly agree.” To reduce response bias, some items were reverse-scored. The total score for each dimension was obtained by summing the scores of the corresponding items, providing a comprehensive assessment of participants’ personality traits. The internal consistency reliabilities (Cronbach’s α) for the subscales of the Big Five Personality Inventory were as follows: openness (α = 0.831), neuroticism (α = 0.906), agreeableness (α = 0.817), conscientiousness (α = 0.903), and extraversion (α = 0.891).

#### 2.2.4. Assessment of Curiosity

Curiosity was assessed using the Curiosity and Exploration Inventory-II (Kashdan et al., 2009). This scale measures two dimensions of curiosity: Stretching and Embracing. Each dimension is assessed by 5 items, for a total of 10 items. The internal consistency coefficient of the scale is 0.85, indicating good reliability. Additionally, the two-factor model demonstrates a good fit, making the scale suitable for assessing curiosity in the context of this study.

### 2.3. Experimental Design

The aim of Study 1 was to explore the relationship between trait creative self-efficacy and creative behavioral performance. Specifically, Study 1 utilized data from 1539 university students. The Alternative Uses Task (AUT) was used to measure performance-based creativity, while the Creative Achievement Questionnaire (CAQ) assessed self-reported creative achievements. The Big Five personality traits and curiosity were included as control variables. Both regression analysis and correlation analysis were conducted to examine the relationship between trait creative self-efficacy and the two forms of creativity.

Building on the findings of Study 1, it was hypothesized that trait creative self-efficacy has a positive effect on creative behavioral performance. The core objective of Experiment 2 was to examine whether controlling participants’ state creative self-efficacy could significantly affect their creative behavioral performance. Since state creative self-efficacy is influenced not only by trait creative self-efficacy but also by external feedback, Experiment 2 employed a 2 × 2 between-subjects design. The two independent variables were “level of trait creative self-efficacy” (high vs. low) and “feedback type” (positive feedback vs. negative feedback). The dependent variables were participants’ state creative self-efficacy and their level of creative behavioral performance. First, participants were grouped into high and low creative self-efficacy categories based on their trait creative self-efficacy scores. Then, within each group, participants were randomly assigned to either the positive feedback or negative feedback condition. A paired sample *t*-test was conducted to analyze whether the scores of the two trait creative self-efficacy groups showed significant differences, ensuring the initial differences between the groups were reasonable.

### 2.4. Experimental Procedure

The detailed procedure of Experiment 2 is as follows. First, participants were recruited through an online questionnaire (Questionnaire 1). The questionnaire included the Trait Creative Self-Efficacy Scale, the Big Five Personality Scale, the Curiosity Scale, the State Creative Self-Efficacy Scale (“I believe I can provide novel answers”), and the Alternative Uses Test (AUT) for creative behavior. Based on the results from Study 1, the openness and neuroticism dimensions from the Big Five Personality Scale, as well as curiosity measured by the Curiosity Scale, were treated as covariates in the regression model. To reduce the Hawthorne effect, a single-blind design was employed.

Before the experiment began, participants were given the following instructions: “This study aims to evaluate a new method of creativity assessment. First, your creativity will be evaluated using a widely accepted existing method, and then your responses on the new measure will be scored based on this evaluation to enhance the accuracy of the new method.” The purpose of these instructions was to mislead participants about the true objective of the experiment, thus minimizing the likelihood that they would guess the actual research purpose and consequently reduce any potential influence of their subjective awareness on the results.

Based on the questionnaire results, participants were divided into two groups: high creative self-efficacy and low creative self-efficacy. The grouping was conducted using a median split (Iacobucci et al., 2015), where participants scoring above the median on the Trait Creative Self-Efficacy Scale were assigned to the high creative self-efficacy group, and those scoring below the median were assigned to the low creative self-efficacy group. After grouping, each participant was randomly assigned to either a positive or negative feedback condition. Participants received 10 s of feedback, which included an evaluation of their level of creativity (high or low). This feedback was used to manipulate their state creative self-efficacy.

Following the feedback, participants completed a second creative behavior test (Questionnaire 2). Before the presentation of the creative tasks (CM), participants were again asked to rate their state creative self-efficacy, which served as a post-test measure for this variable. The experimenter explained that the varying difficulty of the questionnaires might result in participants perceiving their creative performance on the previous test as higher than on the current test, which could affect the accuracy of the creativity ratings. To address this issue, participants’ self-evaluations were used to adjust the final score, ensuring an accurate assessment of the novelty of their responses.

### 2.5. Statistical Analysis

In Study 1, data analysis was primarily conducted using R (version 1.4.1106). First, Pearson correlation analysis was employed to explore the relationship between trait creative self-efficacy and two types of creative behavior. What’s more, MedCalc statistical software(version 22.026) was used to calculate the differences between these correlation coefficients (Schoonjans et al., 1995). Next, the trait creative self-efficacy was used as the independent variable, with creative behavior as the dependent variable and the Big Five personality traits and curiosity as covariates in a linear regression analysis. This aimed to determine the impact of trait creative self-efficacy on both types of creative behavior. Given that this experiment included multiple control variables, to optimize the model and prepare for subsequent experiments, non-significant control variables were removed based on the initial regression results, and a new regression analysis was conducted to generate a model with a better fit. All assumptions for multiple linear regression (independence, normality, homoscedasticity, and absence of multicollinearity) were tested and met, ensuring the validity of the regression analyses.

In Experiment 2, the primary focus was to assess whether the state creative self-efficacy of the two groups improved or declined before and after the experiment, aiming to evaluate the effectiveness of feedback in regulating creative self-efficacy. Due to the differing difficulty levels of the two sets of tasks, the creativity scores of the participants’ creative responses were standardized (Andrade, 2021). After standardization, the post-test scores were subtracted from the pre-test scores to calculate the change in creative behavior. Subsequently, analysis of variance (ANCOVA) was used to examine whether there were differences in the final creative behavior based on different levels of trait creative self-efficacy and feedback types. Based on the findings of Study 1, openness, neuroticism from the Big Five personality traits, and curiosity were included as covariates in the ANCOVA model. Following the ANCOVA results, a paired sample *t*-test was performed to examine the pre-post differences in participants’ state creative self-efficacy and creative behavior, further confirming whether these variables changed because of the type of false feedback and the level of trait creative self-efficacy. Finally, Spearman’s correlation analysis was conducted to verify the relationship between the changes in state creative self-efficacy and creative behavior, addressing whether changes in creative behavior were associated with the changes in state creative self-efficacy.

## 3. Results

### 3.1. The Relationship Between Trait Creative Self-Efficacy and Creative Behavioral Performance

Study 1 employed Pearson correlation analysis and linear regression models to examine the strength of the association between trait creative self-efficacy and two types of creative behavioral performance.

Pearson correlation analysis revealed that the correlation coefficient between trait creative self-efficacy and AUT was 0.100, with *p* < 0.001 and a 95% confidence interval of [0.054, 0.152]. In contrast, the correlation coefficient between trait creative self-efficacy and CAQ was 0.438, with *p* < 0.001 and a 95% confidence interval of [0.403, 0.476]. Since the confidence intervals of these two coefficients do not overlap, and the correlation between trait creative self-efficacy and CAQ is significantly stronger than that with AUT, a comparison of the correlation coefficients using MedCalc indicated a difference of 0.338, Z = 10.238, *p* < 0.001 (as shown in Table 1). This comparison was performed using Fisher’s Z-test, which involves transforming the Pearson correlation coefficients (r1 and r2) into Fisher’s Z-values first (Jiang, 2010). The difference between the two Z-values (Z1 and Z2) was then calculated and tested for statistical significance using the following formula: Z_diff_ = (Z1 − Z2)/sqrt ((1/(n1 − 3)) + (1/(n2 − 3))). Consequently, it can be concluded that the relationship between trait creative self-efficacy and CAQ is significantly stronger than that with AUT.

**Figure 1:**
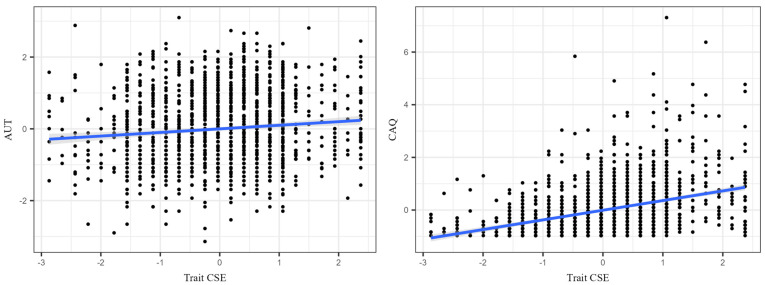
The relationship between trait creative self-efficacy and creative behavior.

In further analyses, trait creative self-efficacy was used as the independent variable, with AUT scores and CAQ scores as dependent variables, respectively. Gender and age were included as covariates to account for potential demographic variability. Research indicates that creativity and creative self-efficacy may vary across gender and age groups due to sociocultural or developmental factors (Abraham, 2016; Jones & Weinberg, 2011). Including these variables ensures that our findings remain robust and generalizable across different populations. Trial was included as a covariate because our data were collected over multiple recruitment time points as part of the large-scale Project (BBP), spanning several years (e.g., 2017, 2018). Variability in temporal context, procedural adjustments, or sample characteristics across sessions could introduce noise. Controlling for the trial ensures that the observed effects are not confounded by such differences, enhancing the robustness of our findings. The results showed that the regression relationships between trait creative self-efficacy and both CAQ and AUT were significant. Specifically, the adjusted R^2^ for the trait creative self-efficacy-CAQ model was 0.1946, *p* < 0.001, while the adjusted R^2^ for the trait creative self-efficacy-AUT model was 0.3488, *p* = 0.01 (see Figure 1). In the regression model for CAQ, the Big Five traits of openness, neuroticism, and agreeableness were significant covariates, while conscientiousness, extraversion, and curiosity were not. In the regression model for AUT, openness, neuroticism, and curiosity were significant, while conscientiousness, extraversion, and agreeableness were not (see Table 2).

To further simplify the models and clarify the impact of significant covariates, Study 1 excluded the covariates that were non-significant in both models, namely conscientiousness and extraversion. After refitting the models, it was found that openness, neuroticism, and curiosity remained significant in both models, while agreeableness continued to be significant in the CAQ model but remained non-significant in the AUT model (see Table 3). Moreover, trait creative self-efficacy continued to have a significant effect on both CAQ and AUT in the new models. The adjusted R^2^ values for the revised models were 0.1952 and 0.3494, respectively, slightly higher than the original models, indicating improved model fit.

In conclusion, this study reveals the complex relationship between trait creative self-efficacy and two types of creative behavioral performance, particularly demonstrating a stronger association between trait creative self-efficacy and CAQ. By excluding non-significant covariates, the optimized regression models provide a clearer explanatory framework, offering insights into the factors that contribute to individual differences in creative behavior.

The scatterplots depict the relationship between trait creative self-efficacy (CSE) and two creative performance measures: Alternative Uses Task (AUT) on the left and Creative Achievement Questionnaire (CAQ) on the right. Each point represents an individual participant, with the blue line indicating a linear trend in each case. The plot on the left shows a weak positive relationship between the trait CSE and AUT scores, while the plot on the right illustrates a stronger positive relationship between the trait CSE and CAQ scores.

### 3.2. The Influence of State Creative Self-Efficacy on Creative Behavioral Performance

#### 3.2.1. Validity Test of Random Grouping

In Experiment 2, participants were randomly assigned to either a high-feedback or low-feedback group before receiving different types of feedback in order to eliminate the potential influence of trait creative self-efficacy on the experimental results. To validate the effectiveness of random grouping, we conducted a difference test on trait creative self-efficacy between the two feedback groups. Furthermore, to ensure that the trait creative self-efficacy grouping was effective, independent sample *t*-tests were conducted. These tests included four key comparisons: the difference in trait creative self-efficacy between the high-feedback and low-feedback groups; the difference in trait creative self-efficacy between the trait-based high and low self-efficacy groups; the difference between high and low trait creative self-efficacy within the high-feedback group; and the difference between high and low trait creative self-efficacy within the low-feedback group (see Table 4).

The results showed no significant difference in trait creative self-efficacy between the high-feedback and low-feedback groups, indicating that the random grouping into feedback conditions was effective. Meanwhile, significant differences were found between the high and low trait creative self-efficacy groups within the same feedback conditions, confirming the validity of the trait creative self-efficacy grouping.

#### 3.2.2. The Impact of Experimental Control on State Creative Self-Efficacy

In Experiment 2, state creative self-efficacy was measured twice using questionnaires. The hypothesis was that the level of state creative self-efficacy in the second questionnaire would be based on the first and modulated by the type of feedback received. Specifically, participants in the high-feedback group were expected to show an increase in creative behavioral performance in the second measurement, whereas those in the low-feedback group were expected to show a decrease. However, as state creative self-efficacy may also be influenced by trait creative self-efficacy and other individual traits, individuals with high trait creative self-efficacy might be less affected by external feedback. Therefore, we used analysis of variance (ANOVA) to examine the effects of trait creative self-efficacy and feedback type on the changes in state creative self-efficacy, with openness, neuroticism, and curiosity as control variables.

**Figure 2:**
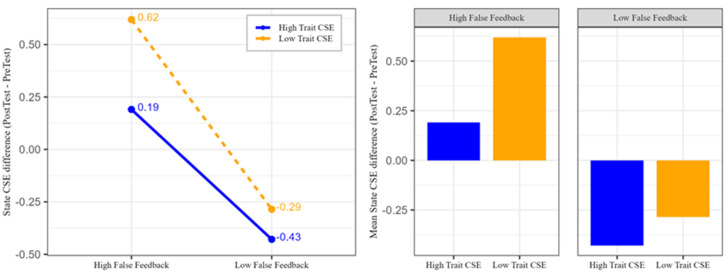
The effects of trait creative self-efficacy and feedback type on the state creative self-efficacy differences (Post-test–Pre-test). The left panel shows the interaction effect of feedback type and trait CSE, with blue representing high trait CSE and orange representing low trait CSE. The right panel shows the mean difference in state CSE for each feedback and trait CSE condition. The dashed lines and bars indicate low trait CSE, while the solid lines and bars indicate high trait CSE. The numbers next to the data points represent the actual mean differences.

As shown in Table 5, feedback type had a significant effect on state creative self-efficacy. Figure 2 illustrates that participants in the high-feedback group exhibited an increase in state creative self-efficacy (positive difference), while those in the low-feedback group showed a decrease (negative difference). However, trait creative self-efficacy did not significantly influence the change in state creative self-efficacy, nor was there an interaction between trait creative self-efficacy and feedback type.

To further verify the effectiveness of feedback in modulating state creative self-efficacy, paired sample *t*-tests were performed, assuming no influence of trait creative self-efficacy on state creative self-efficacy. The precondition for using paired sample *t*-tests is the correlation between the two sets of data. As shown in Table 6, significant correlations were found between pre- and post-test state creative self-efficacy in both the high-feedback and low-feedback groups (*p* < 0.001).

The results of the paired sample *t*-tests indicated that post-test state creative self-efficacy was significantly lower than the pre-test levels in the low-feedback group, confirming that low feedback successfully reduced participants’ state creative self-efficacy. In contrast, post-test state creative self-efficacy was significantly higher than the pre-test levels in the high-feedback group, demonstrating that high feedback effectively enhanced participants’ state creative self-efficacy.

#### 3.2.3. The Impact of Experimental Control on Creative Behavior Performance

In Experiment 2, creative behavioral performance was measured using three dimensions: originality, fluency, and flexibility. Similar to the analysis of state creative self-efficacy, ANOVA was used, with feedback type and trait creative self-efficacy as independent variables, and openness, neuroticism, and curiosity as control variables. The three dimensions of creative behavioral performance served as dependent variables.

**Figure 3:**
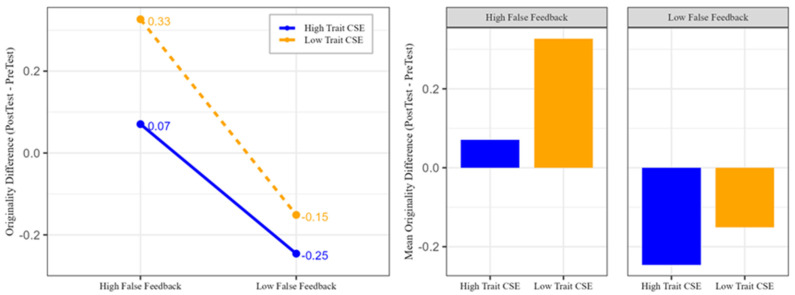
The effects of trait creative self-efficacy and feedback type on originality differences (Post-test–Pre-test). The left panel illustrates the interaction between trait CSE and feedback type, with high trait CSE represented by the solid blue line and low trait CSE by the dashed orange line. The right panel presents the mean originality differences for each combination of feedback type and trait CSE. The blue bars represent high trait CSE, while the orange bars represent low trait CSE. The numbers next to the data points in the left panel indicate the mean differences in the originality scores.

As indicated in Table 7, only originality was significantly affected by feedback type, while fluency and flexibility showed no significant changes. This result parallels the findings for state creative self-efficacy, where originality was influenced solely by feedback type and not by trait creative self-efficacy or interaction effects. Figure 3 further illustrates that participants’ originality increased under high-feedback conditions and decreased under low-feedback conditions, independent of trait creative self-efficacy.

To further verify the effect of feedback on creative behavioral performance, paired sample *t*-tests were conducted to examine the changes in creative behavioral performance before and after feedback. As shown in Table 8, significant correlations were found between the pre- and post-feedback originality levels in both the high-feedback and low-feedback groups (*p* < 0.001).

The results of the paired sample *t*-tests showed that creative behavioral performance in the low-feedback group did not significantly change after receiving feedback, indicating that although state creative self-efficacy decreased, this decline was not sufficient to affect creative behavior. In contrast, the high-feedback group showed a significant increase in creative behavioral performance, suggesting that high feedback not only enhanced state creative self-efficacy but also improved creative behavior.

#### 3.2.4. Relationship Between Changes in State Creative Self-Efficacy and Changes in Creative Behavior Performance

As shown in Table 9, there was no significant correlation between the changes in state creative self-efficacy and any of the three dimensions of creative behavioral performance in the low-feedback group. However, in the high-feedback group, the changes in state creative self-efficacy were significantly correlated with all three dimensions of creative behavioral performance.

In summary, these results indicate that under high-feedback conditions, an increase in state creative self-efficacy can enhance creative behavioral performance across multiple dimensions of creativity.

## 4. Discussion

The present study investigated the relationship between creative self-efficacy and creative performance through two experiments and sought to manipulate state creative self-efficacy using false feedback to improve participants’ creative output. The findings hold significant theoretical and practical implications. First, Study 1 confirmed the independent predictive role of trait creative self-efficacy on creative behavior, demonstrating that even when controlling for variables such as openness, neuroticism, and curiosity from the Big Five personality traits, trait creative self-efficacy remained significantly associated with creative behavior. This finding provides a new perspective for previous research focusing on the relationship between creativity and other personality traits, further confirming the independent role of creative self-efficacy. Experiment 2 revealed the regulatory effects of false feedback on state creative self-efficacy and creative behavior. High creativity feedback significantly enhanced individuals’ state creative self-efficacy and originality levels, validating the applicability of Bandura’s self-efficacy theory in the field of creativity. This discovery offers new insights into improving individuals’ creative performance through external interventions, particularly regarding the potential for positive feedback applications in educational and workplace settings.

In contrast to some previous studies, early research indicated that trait creative self-efficacy was either unrelated or weakly related to performance-based creativity (Karwowski, 2011; Richter et al., 2012), while showing a significant positive correlation with self-reported creative achievements (Chuang et al., 2010). Study 1 supported the finding that trait creative self-efficacy is related to both CAQ and AUT (Haase et al., 2018). The study further demonstrated that trait creative self-efficacy is associated with both CAQ and AUT, possibly suggesting that creative performance may relate to individuals’ beliefs in their creative output (Tierney & Farmer, 2002). Nevertheless, Study 1 found that the correlation between trait creative self-efficacy and CAQ was stronger than its correlation with AUT. Trait creative self-efficacy is a cognitive evaluation reflecting individuals’ confidence in their creative abilities (Karwowski, 2009, 2014). CAQ is a self-assessment measure that may reflect personal self-perception. In Study 1, CAQ was designed to assess actual achievements that may yield over- or under-estimations due to biases in self-perception since the assessment is self-reported. On the other hand, AUT requires individuals to produce works that are both novel and substantial. Higher trait creative self-efficacy may strengthen individuals’ determination to overcome challenges and motivate sustained effort, but the actual creative output is not solely determined by self-efficacy. Moreover, individuals’ self-assessment of their abilities is not always aligned with their actual abilities, which may explain the weaker correlation between trait creative self-efficacy and AUT. The Alternative Uses Task (AUT), for example, primarily measures verbal creativity, specifically divergent thinking (J. Guilford, 1950). Although divergent thinking is a core component of creativity, it does not fully capture an individual’s overall creative performance.

In addition to identifying the relationship between trait creative self-efficacy and creative behavior, Study 1 also incorporated factors closely related to creative self-efficacy and creativity into the regression model as covariates to eliminate their potential effects on this relationship. The results indicated that for CAQ, trait creative self-efficacy was significantly correlated with openness, neuroticism, and agreeableness from the Big Five personality traits. For AUT, openness, neuroticism, and curiosity all showed significant effects. First, this demonstrates that trait creative self-efficacy was significantly associated with creative behavior, independent of other personality traits. In many studies, the Big Five personality traits are not only related to creative self-efficacy (Karwowski & Lebuda, 2016; Karwowski et al., 2013) but are also strongly associated with creativity (Feist, 1998; Furnham & Bachtiar, 2008), particularly openness (George & Zhou, 2001). After including these variables in the regression model, the effect of trait creative self-efficacy on creativity remained significant, indicating that it exerts a unique effect on creativity beyond the indirect effects of personality traits. Second, these findings highlight the importance of controlling personality factors and curiosity in future research to enhance the robustness of the conclusions. Finally, curiosity was significant in the AUT model but not in the CAQ model. This may be because curiosity drives individuals to engage more in exploratory behaviors (Schutte & Malouff, 2020a), which are more likely to result in tangible outcomes that can be objectively evaluated.

Building on these findings, Experiment 2 revealed the significant role of false feedback in regulating state creative self-efficacy. As Bandura pointed out (Bandura, 1977), the sources of self-efficacy include direct experiences, vicarious experiences, verbal persuasion, and emotional arousal. False feedback, presented as an “expert” evaluation, not only elicited emotional responses from participants but also influenced their self-efficacy through verbal persuasion. This study, by regulating creative self-efficacy, was the first to verify the mechanism by which false feedback affects creative performance, particularly demonstrating that high false feedback significantly enhanced participants’ state creative self-efficacy and originality. This finding aligns with previous research on the regulation of cultural self-efficacy through false feedback (Camargo et al., 2020) and provides new evidence for the cross-domain applicability of Bandura’s self-efficacy theory (Bandura, 1986). Notably, it was found that trait creative self-efficacy did not significantly moderate the changes in state creative self-efficacy, which may be attributed to the strong effect of false feedback. High false feedback not only triggered emotional responses but may also have reinforced participants’ self-perception through the immediate positive evaluation, potentially outweighing the enduring effect of trait efficacy on behavior. Moreover, previous research has shown that the immediacy of feedback has a more pronounced effect on short-term behavior regulation, whereas personality traits typically play a dominant role in long-term behavioral outcomes. Therefore, future research could employ longitudinal designs or repeated measurements to further explore the interaction between trait and state self-efficacy and their long-term effects on creative behavior.

The key finding from Experiment 2 was that high levels of false feedback significantly enhanced both participants’ state creative self-efficacy and originality. As a central aspect of creative behavior, originality reflects an individual’s ability to generate novel and unique responses (Chu & Lin, 2013; J. P. Guilford, 1967). The results demonstrated that high false feedback enabled participants to surpass their perceived limitations and successfully complete tasks they initially found difficult. Moreover, this positive feedback stimulated participants’ imagination, encouraging them to express ideas they would typically withhold in everyday situations—precisely the type of high-originality responses targeted in tasks such as the AUT (Alternative Uses Task) and CM (Creative Metaphor) tasks. These results highlight the potential of external reinforcement, such as praise or inflated evaluations, in promoting creativity. This has important practical implications, suggesting that educators and parents can more effectively foster children’s creative performance through positive feedback rather than criticism. Research indicates that criticism often raises stress levels, which can hinder learning outcomes (Flinn et al., 2016), while encouragement effectively boosts motivation (Hermes et al., 2021). Although this study did not directly investigate this mechanism, prior research suggests that increases in self-efficacy lead individuals to set higher goals and persist longer when facing challenges (Haase et al., 2018; Intasao & Hao, 2018), ultimately resulting in enhanced creative performance.

Furthermore, in Experiment 2, although participants in the low false feedback group experienced a decrease in state creative self-efficacy, their originality did not show a significant decline. This result contradicts the initial hypothesis but may reflect the complex psychological mechanisms at play when individuals face negative feedback. Some participants might have experienced a reactive response to low false feedback, attempting to demonstrate their abilities through higher creative performance, while others may have maintained stable levels of creativity based on their self-perception despite receiving negative feedback. This aligns with previous research suggesting that individuals are more likely to accept positive feedback and reject negative feedback (Esdale et al., 2015). These findings imply that creative behavior may be regulated by multiple psychological factors (Dahling & Ruppel, 2016; Lipnevich et al., 2021) rather than being solely dependent on the level of self-efficacy. Future research should introduce additional potential variables, such as reactance, goal orientation, or emotional regulation ability, to further explore the complex mechanisms underlying creative behavior.

The lack of a significant effect of false feedback on fluency and flexibility in creative behavior may be related to the experimental design and time constraints. Fluency and flexibility are primarily associated with the quantity of responses (J. Guilford, 1950; J. P. Guilford, 1967), and previous research has shown that cognitive stimulation can effectively enhance these dimensions (Fink et al., 2010; Fink et al., 2012; Sun et al., 2016). However, the present study did not incorporate cognitive stimulation to broaden participants’ thinking but rather focused on boosting their confidence. This result suggests that future research should integrate different types of task designs, such as tasks with higher cognitive load or those requiring more divergent thinking, to comprehensively assess the impact of false feedback on various dimensions of creative performance. Beyond that, the short duration of the experiment may have led to a ceiling effect, where some participants’ responses approached the upper limits of their cognitive capacity, thereby failing to fully capture the effects of false feedback on fluency and flexibility. Therefore, future studies could extend the experimental duration or incorporate more diverse tasks to explore the broader influence of false feedback on creative performance.

Finally, the practical implications of this study merit further exploration. The findings indicate that positive false feedback can effectively enhance individuals’ creative self-efficacy and promote originality, offering valuable insights for both educational and workplace settings. In education, carefully designed positive feedback mechanisms can help students overcome creative blocks and build confidence in their creative abilities (Boaler, 2022). For instance, formative assessments with constructive feedback could encourage divergent thinking and originality, while activities such as group brainstorming or creative challenges may temporarily boost state creative self-efficacy, enhancing classroom engagement and fostering innovation. Similarly, in workplace management, supportive and affirmative feedback can reinforce employees’ belief in their creative potential and foster a culture of creativity (Dobbs, 1996). Recognizing small wins in team projects or providing structured feedback sessions can stimulate both individual and collective creativity. These findings align with organizational behavior research, which suggests that environments emphasizing psychological safety and positive reinforcement are more likely to yield innovative outcomes, thereby improving team dynamics, employee satisfaction, and overall workplace innovation. However, while positive feedback is effective in the short term, it is equally important to balance it with constructive critiques that are framed to motivate rather than demoralize, ensuring sustained improvements in creative behavior. Future research could further explore how to optimize feedback strategies across diverse contexts, including education and business, to maximize their long-term impact.

Despite the meaningful findings of this study, several limitations remain. First, the study primarily employed correlational and regression analyses. While these analyses verified the relationship between creative self-efficacy and creative behavior, they did not establish clear causal links. Future research should adopt longitudinal designs or experimental manipulations to further validate the causal relationship between the two. Second, the selection of only right-handed participants in Sample 2 represents a methodological limitation. While this decision was made to minimize variability associated with hemispheric lateralization and ensure greater internal validity, it limits the generalizability of the findings to left-handed individuals. Research suggests that handedness may influence cognitive processes, including creativity, with some studies indicating higher levels of creativity in left-handed individuals (Rahimi, 2023; Papadatou-Pastou, 2018; Lindell, 2011). Future studies should include participants with diverse handedness and explore the potential moderating effects of handedness on creative self-efficacy and performance. Finally, this study did not fully consider other potential moderating factors in false feedback, such as individuals’ learning goals, negative emotions, or self-regulation strategies. These factors may be associated with variations in how feedback functions in practical settings, and future research should incorporate additional variables to thoroughly examine the mechanisms underlying feedback regulation.

In conclusion, this study offers a comprehensive examination of creative self-efficacy, elucidating the pivotal roles that both trait and state creative self-efficacy play in shaping creative behavior. By expanding the theoretical framework established by prior research, this work provides a more nuanced understanding of the dynamic interplay between creative self-efficacy and creativity while also opening new avenues for future empirical investigations. Furthermore, the findings underscore the practical implications of feedback as a regulatory mechanism for enhancing creative performance, presenting both significant theoretical advancements and valuable insights for applied contexts.

## Figures and Tables

**Table 1:** Correlations and comparison between trait creative self-efficacy (CSE) and creative performance.

Variable	Correlation Coefficient (r)	95% Confidence Interval (CI)	*p*-Value
CAQ	0.438	[0.403, 0.476]	< 0.001
AUT	0.100	[0.054, 0.152]	< 0.001
**Comparison**	**Difference in Correlation (Δr)**	**Z-Value**	***p*** **-Value**
CAQ vs. AUT	0.338	10.238	<0.001

**Table 2:** Regression models for trait creative self-efficacy predicting creativity.

Predictor	Beta	SE	t	*p*
**AUT**				
CSE	0.079	0.028	2.795	0.005
Sex	0.091	0.022	4.237	<0.001
Age	−0.012	0.021	−0.557	0.578
Time	0.569	0.021	27.061	<0.001
Openness	0.096	0.024	3.943	<0.001
Neuroticism	0.07	0.025	2.824	0.005
Extraversion	−0.017	0.025	−0.678	0.498
Agreeableness	−0.005	0.022	−0.213	0.832
Conscientiousness	−0.002	0.025	−0.085	0.932
Curiosity	0.065	0.028	2.293	0.022
**CAQ**				
CSE	0.279	0.031	8.849	<0.001
Sex	0.118	0.024	4.909	<0.001
Age	0.008	0.023	0.33	0.742
Time	0.009	0.023	0.38	0.704
Openness	0.184	0.027	6.82	<0.001
Neuroticism	0.114	0.028	4.125	<0.001
Extraversion	0.001	0.028	0.046	0.963
Agreeableness	−0.069	0.025	−2.765	0.006
Conscientiousness	0.026	0.027	0.946	0.344
Curiosity	0.061	0.032	1.937	0.053

The reported coefficients (Beta) are standardized regression coefficients, which indicate the strength and direction of the relationship between the predictors and the outcome variables.

**Table 3:** New regression models for trait creative self-efficacy predicting creativity.

Predictor	Beta	SE	t	*p*
**AUT**				
CSE	0.077	0.028	2.775	0.006
Sex	0.09	0.021	4.207	<0.001
Age	−0.012	0.021	−0.561	0.575
Time	0.569	0.021	27.082	<0.001
Openness	0.093	0.024	3.909	<0.001
Neuroticism	0.075	0.023	3.296	0.001
Agreeableness	−0.007	0.022	−0.315	0.753
Curiosity	0.06	0.027	2.189	0.029
**CAQ**				
CSE	0.283	0.031	9.126	<0.001
Sex	0.12	0.024	5.033	<0.001
Age	0.008	0.023	0.32	0.749
Time	0.008	0.023	0.357	0.721
Openness	0.187	0.027	7.037	<0.001
Neuroticism	0.106	0.025	4.156	<0.001
Agreeableness	−0.064	0.024	−2.646	0.008
Curiosity	0.063	0.03	2.096	0.036

The reported coefficients (Beta) are standardized regression coefficients, which indicate the strength and direction of the relationship between the predictors and the outcome variables.

**Table 4:** Independent *t*-test results for trait creative self-efficacy (CSE) across different feedback and CSE levels.

Comparison Groups	Mean Score 1	Mean Score 2	*p*-Value
High Feedback vs. Low Feedback	19.548	20.238	0.44
High Trait CSE vs. Low Trait CSE	23.024	16.762	<0.001 ***
High Feedback: High Trait CSE vs. Low Trait CSE	22.667	16.429	<0.001 ***
Low Feedback: High Trait CSE vs. Low Trait CSE	23.381	17.095	<0.001 ***

*p* < 0.001 (***).

**Table 5:** ANOVA results for state creative self-efficacy (CSE).

Independent Variables	*p*-Value	Adjusted R^2^
Trait CSE	0.153	0.185
Feedback Type	<0.001 ***	
Trait CSE × Feedback Type	0.389	
**Control Variables**		
Openness	0.274	
Neuroticism	0.832	
Curiosity	0.542	

*p* < 0.001 (***); Adjusted R^2^ represents the overall model fit.

**Table 6:** Paired sample *t*-test results for state creative self-efficacy (CSE).

Group	Pre-Test Mean	Post-Test Mean	Difference *p*-Value	Correlation Coefficient	Correlation *p*-Value	Cohen’s d
High False Feedback	2.857	3.262	0.001 **	0.593	<0.001 ***	0.701
Low False Feedback	3.143	2.786	0.009 **	0.553	<0.001 ***	0.850

*p* < 0.01 (**), *p* < 0.001 (***).

**Table 7:** ANOVA results for creative performance behaviors.

Dependent Variable	Independent Variables	*p*-Value	Adjusted R^2^
**Originality**	Trait CSE	0.396	0.061
	Feedback Type	0.009 *	
	Trait CSE × Feedback Type	0.563	
	**Control Variables**		
	Openness	0.889	
	Neuroticism	0.189	
	Curiosity	0.565	
**Fluency**	Trait CSE	0.471	0.027
	Feedback Type	0.179	
	Trait CSE × Feedback Type	0.880	
	**Control Variables**		
	Openness	0.889	
	Neuroticism	0.256	
	Curiosity	0.865	
**Flexibility**	Trait CSE	0.580	0.027
	Feedback Type	0.273	
	Trait CSE × Feedback Type	0.742	
	**Control Variables**		
	Openness	0.451	
	Neuroticism	0.266	
	Curiosity	0.503	

*p* < 0.05 (*). Adjusted R^2^ represents the proportion of variance explained by the independent variables.

**Table 8:** Paired sample *t*-test results for originality (standardized scores).

Group	Pre-Test Mean (Standardized)	Post-Test Mean (Standardized)	Difference *p*-Value	Correlation Coefficient	Correlation *p*-Value	Cohen’s d
High False Feedback	0.108	0.306	0.037 *	0.793	<0.001 **	0.598
Low False Feedback	–0.306	–0.108	0.075	0.607	<0.001 **	0.704

*p* < 0.05 (*), *p* < 0.01 (**).

**Table 9:** Correlations between state creative self-efficacy (CSE) and three subdimensions of creative performance.

Group	Creative Dimension	Correlation Coefficient (r)	*p*-Value
**Low False Feedback**	Originality	0.202	0.200
	Fluency	0.236	0.133
	Flexibility	0.218	0.165
**High False Feedback**	Originality	0.656	<0.001 **
	Fluency	0.508	0.001 *
	Flexibility	0.567	<0.001 **

*p* < 0.05 (*), *p* < 0.01 (**).

## Data Availability

The data used in this study are available from the corresponding author upon reasonable request.
